# Structure of Metaphase Chromosomes: A Role for Effects of Macromolecular Crowding

**DOI:** 10.1371/journal.pone.0036045

**Published:** 2012-04-23

**Authors:** Ronald Hancock

**Affiliations:** Laval University Cancer Research Centre, Hôtel-Dieu Hospital, Québec, Québec, Canada; The Scripps Research Institute, United States of America

## Abstract

In metaphase chromosomes, chromatin is compacted to a concentration of several hundred mg/ml by mechanisms which remain elusive. Effects mediated by the ionic environment are considered most frequently because mono- and di-valent cations cause polynucleosome chains to form compact ∼30-nm diameter fibres in vitro, but this conformation is not detected in chromosomes in situ. A further unconsidered factor is predicted to influence the compaction of chromosomes, namely the forces which arise from crowding by macromolecules in the surrounding cytoplasm whose measured concentration is 100–200 mg/ml. To mimic these conditions, chromosomes were released from mitotic CHO cells in solutions containing an inert volume-occupying macromolecule (8 kDa polyethylene glycol, 10.5 kDa dextran, or 70 kDa Ficoll) in 100 µM K-Hepes buffer, with contaminating cations at only low micromolar concentrations. Optical and electron microscopy showed that these chromosomes conserved their characteristic structure and compaction, and their volume varied inversely with the concentration of a crowding macromolecule. They showed a canonical nucleosomal structure and contained the characteristic proteins topoisomerase IIα and the condensin subunit SMC2. These observations, together with evidence that the cytoplasm is crowded in vivo, suggest that macromolecular crowding effects should be considered a significant and perhaps major factor in compacting chromosomes. This model may explain why ∼30-nm fibres characteristic of cation-mediated compaction are not seen in chromosomes in situ. Considering that crowding by cytoplasmic macromolecules maintains the compaction of bacterial chromosomes and has been proposed to form the liquid crystalline chromosomes of dinoflagellates, a crowded environment may be an essential characteristic of all genomes.

## Introduction

Metaphase chromosomes are formed by two giant polynucleosome chains, one in each chromatid and 1.7–8.5 cm long in human cells, compacted to a measured average density of several hundred mg/ml [1], [2] consistent with values calculated from their DNA content and volume [3], [4]. The conformation of the polynucleosome chains and the mechanism(s) by which this dense packing is achieved are not understood. The primary contribution is generally believed to be from electrostatic effects mediated by interactions of monovalent and/or divalent cations, principally K^+^, Na^+^, and/or Mg^2+^, because in vitro these cations cause polynucleosomes to fold to a compact helical conformation termed the 30-nm fibre [5]–[7], and media containing these cations at millimolar concentrations, often with the polycations spermine and/or spermidine, are usually used to isolate chromosomes [8]–[12]. Chromatin fibres of ∼30 nm diameter cannot be detected in chromosomes in situ [13], however, suggesting that other factors may contribute to the dense packing of chromatin in chromosomes in vivo.

A further parameter which has not been considered is predicted to influence strongly the structure of chromosomes in vivo, namely the high concentration of macromolecules in the cytoplasm surrounding them after the nuclear envelope is disassembled in prophase. The cytoplasm of mitotic cells contains proteins at ∼105 mg/ml together with RNA at ∼42 mg/ml according to in situ studies [2], consistent with evidence that its concentration of macromolecules is similar to that of the cytoplasm in interphase [14] which has been measured to be 130–200 mg/ml of diffusible macromolecules [15]–[17]. In these highly crowded conditions within and outside chromosomes the close proximity of macromolecules results in strong attractive forces, termed entropic or depletion forces, between them [18]–[20], and it has been amply demonstrated that linear polyelectrolyte polymers [21], [22] including DNA [23] and polynucleosomes [24] adopt collapsed, compact conformations in similar conditions. The chromosome of *Escherichia coli* is maintained in its compact conformation in vivo due to crowding by cytoplasmic macromolecules, and its compaction is conserved in vitro if an inert volume-occupying macromolecule is included in the medium to reproduce this crowding [20]. It is notable that in these conditions, the divalent cations and/or polyamines which were used earlier to stabilise these chromosomes are no longer required [20]. Here, in experiments aimed to examine if the packing of chromatin in metaphase chromosomes could be influenced by the crowding effects of cytoplasmic macromolecules, chromosomes were found to conserve their characteristic structure when they were isolated in media containing an inert, volume-occupying macromolecule (polyethylene glycol, dextran, or Ficoll) without significant concentrations of exogenous ions and with no polyamines. These findings suggest that crowding effects due to cytoplasmic macromolecules may play a significant role in determining the compact structure of the genome in metaphase chromosomes.

## Results

### Isolation of chromosomes in medium containing a crowding macromolecule

Chromosomes were released from mitotic chinese hamster ovary (CHO) fibroblasts by disrupting them in a solution containing a volume-occupying macromolecule of the type which is widely employed to study crowding effects in vitro [25]–[28]. The macromolecules used were polyethylene glycol (PEG) (*M*_r_ 8 kDa), dextran (*M*_r_ 10.5 kDa), or Ficoll (*M*_r_ 70 kDa) at a concentration expressed as (w/v), with 100 µM K-Hepes buffer, pH 7.4, as the only supplement. To disrupt mitotic cells, disperse membranes and cytoplasmic material, and release chromosomes these solutions were supplemented with Triton X-100 (0.5% v/v), and the chromosomes were cytocentrifuged onto slides in conditions which reduced the contamination by smaller cellular components to a minimum.

**Figure 1:**
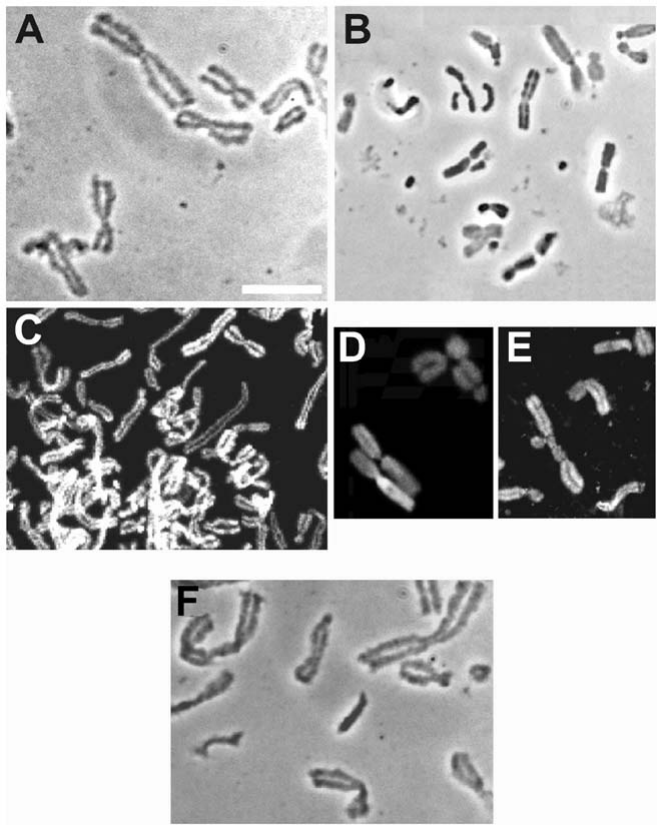
(A–E) Metaphase chromosomes released from mitotic CHO cells in a solution containing a crowding macromolecule in 100 µM K-Hepes buffer. Representative fields of chromosomes cytocentrifuged and fixed in the same medium as that used for cell lysis. (A, B, F) phase-contrast images; (C–E) DNA labeled with YOYO-1. Chromosomes were released in (A) 12% PEG (M_r_ 8 kD); (B) 25% PEG; (C) 20% PEG; (D) 40% Ficoll (M_r_ 70 kD); (E) 12% dextran (M_r_ 10.5 kD). (F) Chromosomes isolated by a conventional method [29] from a sample of the mitotic cells used in panel A. Magnification is the same in all panels; scale bar in A, 5 µm.

Chromosomes released in a solution containing 12% PEG, 12% dextran, or 40% Ficoll conserved the characteristic structure of those isolated by conventional procedures (Figure 1A–E). Their size and compaction showed some variation in solutions containing different concentrations of a crowding macromolecule, an effect which is discussed below. For comparison, Figure 1F shows chromosomes released in a conventional polyamine-containing buffer [29] from a sample of the mitotic cells used in Figure 1A.

This conservation of the characteristic structure of chromosomes in solutions containing 100 µM K-Hepes buffer as the only ionic component contrasted with the large increase in volume of chromosomes isolated by conventional procedures [30]–[32] and of chromosomes in situ [33], [34] in media of low ionic strength. To confirm that their structure was not influenced by contaminating cations in the solutions of crowding macromolecules, these were assayed by atomic emission spectrometry. In a 12% solution of PEG the concentrations were <4 µM Mg^2+^, 1.1 µM Ca^2+^, 18 µM Na^+^, and 710 µM K^+^; most of this K^+^ (∼650 µM) originated from KOH required to neutralise unidentified components in commercial PEG and was not present in solutions of the other crowding macromolecules. In solutions containing cations at these concentrations chromatin fibres and polynucleosomes have an extended conformation, and they become progressively more compact only when the concentration reaches ∼60 mM for Na^+^ or ∼0.3 mM for Mg^2+^ [7].

### Structure of chromosomes by electron microscopy

**Figure 2:**
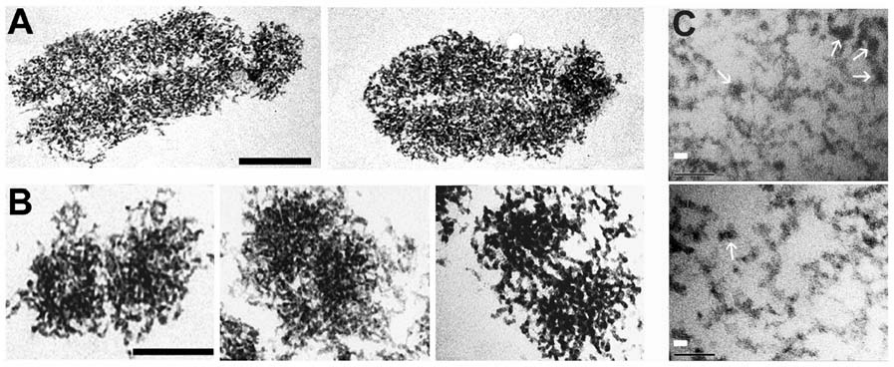
Images by transmission electron microscopy of chromosomes released in 12% PEG. Sections are approximately longitudinal or transversal in (A) and (B), respectively. (C) Chromatin fibres in regions of lower density at the periphery of chromosomes; white arrows illustrate regions where fibres of ∼30 nm diameter are seen. Scale bars (A, B), 1 µm; (C), 30 nm.

Images of chromosomes sectioned for electron microscopy after release in 12% PEG are shown in Figure 2. In general, these images resemble those of chromosomes prepared by other methods [8]–[12]. The diameter of chromosomes measured on longitudinal sections was 1370±85 nm (mean ±SEM, n = 14), larger than that of chromosomes isolated in cation- or polyamine-containing buffers (700–800 nm) [10]. The diameter of individual chromatids from transverse sections (Figure 2B) was 590±40 nm. The dense packing of chromatin fibres precluded reliable measurements of their diameter and tracing their paths, but in less densely-packed regions at the periphery of chromosomes their width was variable and between 10 and 40 nm (Figure 2C).

### Variation of chromosome volume with concentration of a crowding macromolecule

**Figure 3:**
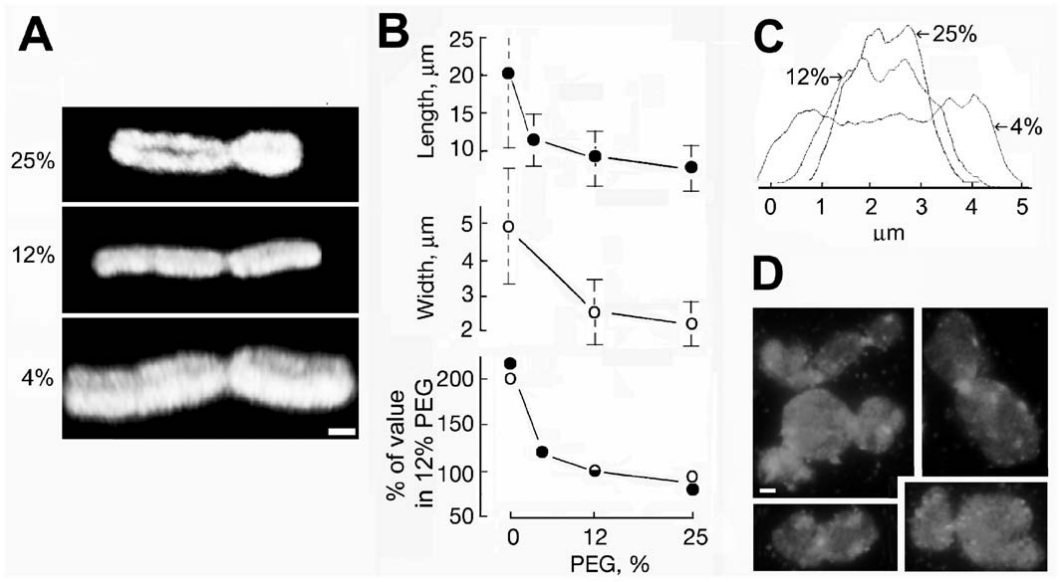
Influence of the concentration of crowding agent on chromosome dimensions. Chromosomes released in 12% PEG were deposited on slides and incubated for 1 h with PEG at the concentration shown in 100 µm K-Hepes buffer, fixed in the same solution, and DNA was labeled with YOYO-1. (A) 3-D volume of the largest chromosome of CHO cells reconstructed from serial confocal sections; scale bar, 1 µm. (B) Length of the largest chromosome, diameter of randomly selected chromosomes, and these values expressed as the % of those in 12% PEG; error bars show SEM from measurements of ≥15 chromosomes. (C) Transverse linescans of fluorescence intensity across representative chomosomes labeled with YOYO-1. (D) Representative images of chromosomes incubated in 100 µm K-Hepes buffer with no PEG for 1 h and labeled with YOYO-1. Scale bar, 1 µm.

The images in Figure 1 show that chromosome dimensions varied with the concentration of crowding macromolecules in the surrounding medium. This effect could be visualised more clearly by reconstructing the 3-D volume of the largest chromosome in the CHO cell karyotype [35], which could be identified unambiguously when the density of chromosomes on slides was low (Figure 3A). Measurements of chromosome width after incubation in different concentrations of PEG, which was relatively constant for chromosomes of all sizes, together with the length of the longest chromosome showed that these dimensions varied approximately isotropically (Figure 3B). Transverse linescans of the fluorescence intensity of YOYO-1-stained chromosomes showed the radial distribution of DNA (Figure 3C), but the limited resolution of optical microscopy was insufficient to detect if a region of lower density existed in the central region of chromatids (∼3% of their width) as predicted by a recent polymer model of chromosomes [36]. Incubation of chromosomes in the absence of a crowding macromolecule resulted in marked expansion, but they did not disperse completely during the incubation time of 1 h (Figure 3D). Together, these observations show that the concentration of crowding macromolecule in the solution was the crucial factor which determined the compaction of isolated chromosomes.

### Nucleosomal structure, topoisomerase IIα, and SMC2 in chromosomes

**Figure 4:**
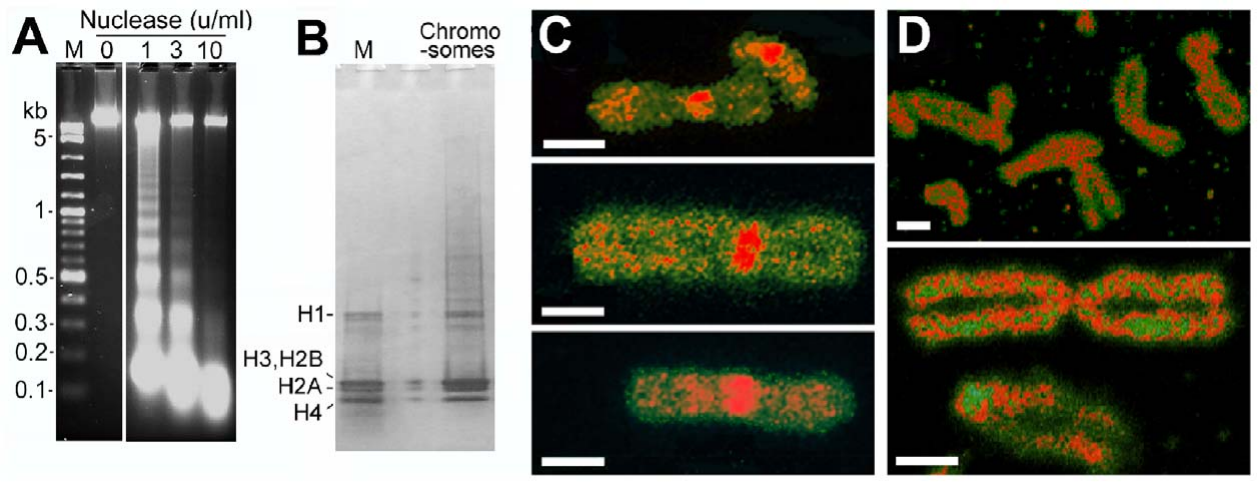
Nucleosomal structure and nonhistone proteins of chromosomes released in 12% PEG. (A) DNA fragments from chromosomes incubated with micrococcal nuclease, separated on a 2% agarose gel; M, length markers. (B) Proteins extracted from chromosomes in 0.2 N H_2_SO_4_ and separated in a 4–20% denaturing SDS-PAGE gel; markers (M) were purified histones from calf thymus. (C) Topoisomerase IIα and (D) SMC2 visualised by immunofluorescence (red); DNA was labeled with YOYO-1 (green). Scale bars, 1 µm.

Chromosomes isolated in 12% PEG and incubated with micrococcal nuclease showed a pattern of nucleosome-protected DNA fragments whose monomer length was initially ∼180 bp (Figure 4A), a value essentially identical to that (177 bp) in chromosomes of CHO cells isolated by a conventional method [37]. As well as a canonical pattern of histones, some larger acid-soluble polypeptides were detectable (Figure 4B); these probably originate from ribosomes and RNP particles since the chromosomes were not purified further after centrifugation from the cell lysate. Topoisomerase IIα and the SMC2 subunit of condensin, which are predominant non-histone proteins in chromosomes isolated by conventional methods [38]–[42], were identified by immunofluorescence (Figure 4C, D). The patterns of labelling of these proteins along the chromatid axes were irregular, like those observed in other studies [38], [41], for reasons which are not clear. Topoisomerase IIα more intense signal in the centromeric region, as observed in other cell types particularly in the prometaphase or metaphase stage [42].

## Discussion

The essential conclusion of these experiments is that the characteristic structure and compaction of metaphase chromosomes are conserved when they are isolated in media which contain a volume-occupying crowding macromolecule, with concentrations of K^+^, Na^+^, Ca^2+^, and Mg^2+^ ions in the low micromolar range. Theory predicts that assemblies of macromolecules are stabilised in crowded conditions [19], [28], and this has been confirmed experimentally in numerous cases including filaments of actin [43] and of tubulin [44], ribosomes [25], oligomers of the chaperonin GroEL [26], HIV capsids [27], bacterial chromosomes [20], and intranuclear structures [45]. The concentration of a crowding macromolecule required to reproduce the compaction of chromosomes in vivo cannot be estimated precisely from the present data, but an approximate value could be deduced from the diameter of chromatids measured by electron microscopy (Figure 2B). In 12% PEG the diameter of chromatids was 590±40 nm (Figure 2B), within the range of values measured for single chromatids in living CHO cells (400–600 nm) [41] and for entire chromosomes in living CHO and NRK cells (∼1 µm) [46], [47]. The osmotic pressure in this solution, which is an alternative manner of viewing macromolecular crowding forces [20], [48], is ∼200 kPa [49] or approximately equivalent to that of a solution containing BSA at ∼200 mg/ml [50].

The conservation of chromosome structure in crowded media in which the concentrations of K^+^, Na^+^, Ca^2+^, and Mg^2+^ ions were 100–1000-fold lower than those usually employed for their isolation, often together with polyamines [8]–[12], is consistent with the elimination of a requirement for ions for stabilisation of other macromolecular assemblies in crowded conditions [25]–[27], [44]. The extent to which ionic conditions in the cell are reproduced by media commonly used to isolate chromosomes is difficult to evaluate; concentrations of diffusible (osmotically active) ions in vivo cannot be derived from measurements of their total quantities because significant fractions of K^+^ and Na^+^ appear to be bound to macromolecules [51]–[55] and of Mg^2+^ to ATP, mitochondria, and the sarcoplasmic reticulum [56], and it has been argued that the cytoplasm contains essentially no free ions [57]. Polyamines at micromolar concentrations cause compaction of chromatin fibres and have significant effects on other properties of chromatin [58], [59], and their effects on the structure of chromosomes merit consideration as noted in [12].

As already emphasised [13], observations on the conformation of chromatin fibres at low concentrations in vitro must be extrapolated with caution to conditions in vivo where the concentration of nucleosomes in chromosomes is vastly higher, resulting in strong entropic inter-fibre attractive forces which create compact conformations resembling a polymer melt [13], [60]. The compaction of linear polymers like polynucleosome chains or DNA by these forces is well established by both simulation and experiments [21]–[24]. A significant contribution to the compaction of polynucleosome chains is likely to be provided by nucleosome-nucleosome interactions, which are sufficiently strong to form liquid crystals in crowded conditions [61], and theory predicts that the fibres formed will be irregular with different degrees of local compaction because polynucleosome chains are mosaics with interspersed repeated DNA sequences, isochores, and nucleosomes with different histone variants and post-translational modifications, like a multiblock polymer [62]. Polymers of appropriate stiffness can adopt compact cylindrical conformations not unlike a metaphase chromatid [63], and recent simulations show dramatically how conformations of this type could be formed by entropically-favoured looping of a chromatin fibre [36].

The concept that entropic forces make crucial contributions to the conformation of chromatin in vivo is not novel, and indeed is central to current models of interphase chromosomes where they contribute to forming chromatin loops [64]–[67] and discrete chromosome territories [68]. These models do not, however, exclude a contribution of electrostatic effects; ions which were strongly bound in chromosomes would not be extracted in the conditions used here, and a subtle interplay is seen between the effects of crowding and electrostatic forces when a polyelectrolyte polymer bearing counterions, a model for a polynucleosome chain, collapses in crowded conditions [21], [69].

The results described here, together with the evidence that macromolecular crowding is a crucial factor in structuring the interphase genome [64], bacterial chromosomes [20], [70], and possibly polytene chromosomes [71] and the liquid crystalline chromosomes of dinoflagellates [72], are consistent with the hypothesis that a crowded environment is an essential characteristic of all genomes. This model has particularly interesting implications for meiotic chromosomes, because pairing of homologous DNAs [73], [74] and recA-promoted exchange of DNA strands [75] are stimulated in crowded conditions.

## Materials and Methods

### Isolation of chromosomes

Mitotic cells were detached from semi-confluent monolayers of CHO cells (CHO-K1, ATCC) growing in McCoy's 5a medium with 10% FCS by shaking horizontally for 2 min after incubation for 2 h with nocodazole (60 ng/ml; Sigma-Aldrich). Cells were centrifuged and resuspended at room temperature in a solution of PEG (average *M*_r_ 8 kDa, Fluka BioUltra), dextran (10.5 kDa, Sigma-Aldrich), or Ficoll (70 kDa, Fluka) in bidistilled H_2_O, deionised by shaking with AG 501-X8 resin (Bio-Rad) for 6–8 h, supplemented with 100 µM K-Hepes buffer, pH 7.4. Before each experiment the pH of these solutions was verified and adjusted to pH 7.4 if neccessary. Cation concentrations in polymer solutions were measured by atomic emission spectrometry (Varian Vista-Pro). Cells were centrifuged (300 *g*, 10 min in 12% PEG or 12% dextran; 500 *g*, 20 min in 40% Ficoll) and resuspended at ∼5×10^6^ cells/ml in the same solution containing 0.5% (v/v) Triton X-100 (Sigma-Aldrich). After 5 min chromosomes were released by ∼50 hand strokes in a 2 ml Teflon-glass homogeniser (Wheaton) and one volume of the same solution without Triton was added with gentle mixing. Chromosomes were also prepared by a conventional procedure for comparison; mitotic cells were homogenised in 7.5 mM Tris-HCI (pH 7.4), 0.1 mM spermine, 0.25 mM spermidine, 1 mM EDTA (pH 7.4) and 40 mM KCI [29], and cytocentrifugation as described below.

### Optical imaging and immunofluorescence

Chromosomes were cytocentrifuged onto polylysine-coated slides (300 *g*, 20 min in PEG and dextran; 500 *g*, 40 min in Ficoll). When indicated, they were overlayed with 500 µl of solution of a crowding macromolecule and incubated in a humidified container for 1 h. Fixation was for 10 min in the same solution as the previous step supplemented with 2% formaldehyde by adding 16% aqueous formaldehyde solution, pH 7.4 (Ted Pella); this fixation was used to immunolabel topoisomerase II and methanol (−20°C, 15 min) for SMC2. Antibodies were rabbit anti-human topoisomerase IIα (Topogen) (1/20, 4 h) or rabbit anti-human SMC2 (Abcam antibody 10399) (1/500, 1 h) followed by Alexa 594-secondary antibody (Invitrogen) (1/500, 1 h). DNA was labeled with YOYO-1 (1 µM, 10 min). Phase-contrast images were acquired with a CoolSNAP camera (Roper Scientific) on a Nikon E800 microscope with a 100× NA 1.3 oil-immersion objective. Confocal images of 0.2 µm sections acquired on an MRC1024 microscope (BioRad) with a 60× NA 1.4 oil-immersion objective were deconvoluted (nearest neighbour) and are shown as maximum intensity projections made with Metamorph 7.65 (Universal Imaging). 3-D volumes were constructed with Volocity 5.4 (PerkinElmer) and dimensions and linescans were made with ImageJ (http://rsb.info.nih.gov/ij; developed by Wayne Rasband, NIH). Grayscale images were pseudocoloured and merged using Photoshop 7.0 (Adobe).

### Transmission electron microscopy

Chromosomes released in 12% PEG solution were centrifuged (700 *g*, 10 min), resuspended in the same solution, and fixed by adding 16% formaldehyde to a concentration of 2% (see above) and glutaraldehyde (Sigma-Aldrich) to 0.1%. After 1 h on ice they were cytocentrifuged onto a 2 µm film of Aclar (EMS) fixed to a slide and the entire sample was detached, dehydrated, and embedded in Poly/Bed 812 (Polysciences). Sections (90–100 µm) cut parallel or perpendicular to the Aclar film were stained with uranyl acetate and lead citrate by standard methods. Digital images were acquired on a Jeol 1200 microscope at 20,000–40,000 magnification.

### Nucleosomal structure

Chromosomes released in 12% PEG solution were centrifuged (500 *g*, 10 min), incubated with micrococcal nuclease at 37°C as described in [37]. and DNA fragments were phenol-extracted and separated on a 2% agarose gel. Histones were extracted from chromosomes in 0.2 N H_2_SO_4_ (30 min, 4°C), precipitated with 80% ethanol, and separated by denaturing SDS-PAGE in a 4–20% gradient gel.
